# Study of the enteric and motor inervation, pelvic musculature, and alterations in the sacral region of rat fetuses with ethylenethiourea-induced anorectal anomaly

**DOI:** 10.1590/acb401525

**Published:** 2025-03-10

**Authors:** Evandro Luis da Cunha Oliveira, Yvone Avalone de Moraes Villela de Andrade Vicente

**Affiliations:** 1 Universidade de São Paulo – Faculty of Medicine of Ribeirão Preto – Department of Surgery – Ribeirão Preto (SP) – Brazil.; 2 Universidad Autónoma de Madrid – Madrid – Spain.; 3 University of Alabama at Birmingham – Birmingham – United States of America.

**Keywords:** Embryonic Structures, Ethylenethiourea, Anus, Imperforate

## Abstract

**Purpose:**

The aims of this work were to induce anorectal anomaly in rat fetuses via the planned administration of ethylenethiourea (ETU), and to study fetuses exhibiting anorectal malformation, as well as apparently normal fetuses submitted to the effect of ETU.

**Methods:**

Time-mated pregnant Wistar rats were randomly divided into control and experimental groups. On gestational day 10, the experimental group received 10% ETU (130 mg/kg) by gavage, whereas the control rats received vehicle only. The embryos were harvested by cesarean section on gestational day 21. The fetuses exposed to ETU were divided into two groups: affected (without any clear anorectal alterations); and the malformed (with anorectal anomaly). The neuromotor plates were identified by immunohistochemistry with acetylcholinesterase, and alterations in the sacral region were evaluated by histological and morphometric studies.

**Results:**

We used 43 control fetuses, 82 affected fetuses, and 118 malformed fetuses in this study. The most frequent associated macroscopic anomalies were spina bifida (55 fetuses), encephalocele (20), and alterations in the lower limbs (5). The sacroiliac was malformed in 45% of the affected fetuses and in 53.2 % of the malformed fetuses.

**Conclusion:**

ETU leads to a reduced number of motor neurons in the pelvic musculature of both the malformed and the affected rats. The enteric neurons are altered in the malformed fetuses, but not in the affected ones. Both the affected and malformed rats exhibit sacral alterations that do not interfere with neurons.

## Introduction

The etiopathogeny of the anorectal anomaly, a congenital malformation whose main feature is the absence or the ectopic location of the anus, is still not clear^1^^–^ 3. The severity of the condition depends on the integrity of the sacroiliac, the musculature, and the neurons, as well as on other associated malformations^4^^–^ 6.

Fifty percent of the patients with anorectal anomaly exhibit alterations in the lumbosacral region^7^. The extension of this malformation bears straight relation with the severity and prognosis of the anomaly. In severe cases, sphincter control is poor because of alterations in the sacral region and in the pelvic musculature^8^^–^ 10. In fact, sphincter control may be also impaired even in patients with less serious malformations due to alterations in the intestinal motility, pelvian musculature, or sacroiliac, which all probably result from a fault in embryo development^11^^,^ 12 ^,^ 13.

Administration of ethylenethiourea (ETU) to pregnant rats induces a high level of anorectal malformation and low fetal reabsorption. Therefore, this is a suitable model not only for the study of fetuses with anorectal anomaly, but also for the study of fetuses that do not exhibit the anomaly despite having been submitted to ETU.

The aims of this work were twofold: to induce anorectal anomaly in rat fetuses via the planned administration of ETU, and to study fetuses exhibiting anorectal malformation, as well as apparently normal fetuses submitted to the effect of ETU.

## Methods

**Figure 1:**
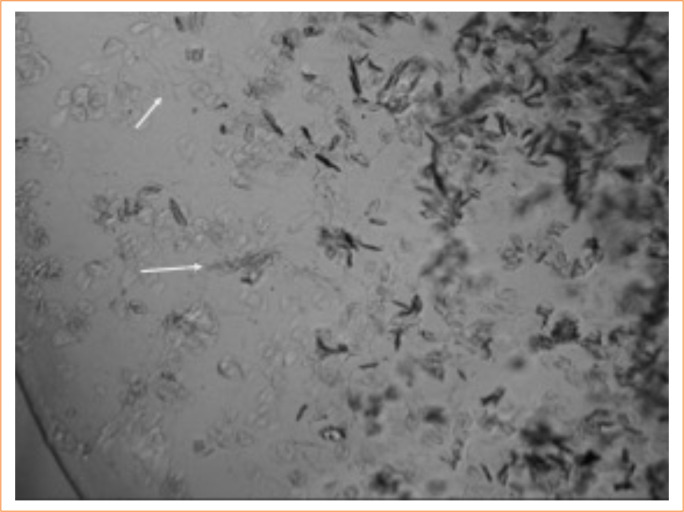
Female rat vaginal smear.

Source: Elaborated by the authors.

ETU (2-Imidazolidinethione, C3H6N2S) powder (Fragon LTDA, São Paulo, SP, Brazil) was dissolved in distilled water to a concentration of 10%. Sixty female Wistar rats (Animal Laboratory, School of Medicine, Ribeirão Preto, SP, Brazil) were time-mated with a male rat overnight. The day of sperm detection in the female rat vaginal smear was designated as gestational day 0 (Fig. 1).

**Figure 2:**
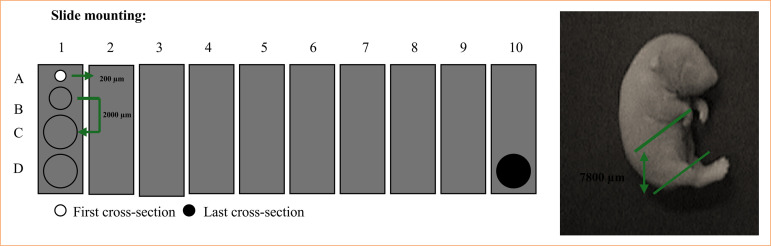
Schematic representation of slide mounting.

Source: Elaborated by the authors.

**Figure 3:**
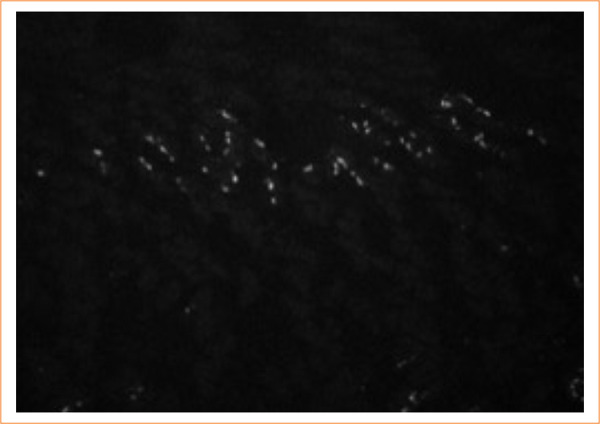
Immunefluorescence of the rat musculature.

Source: Elaborated by the authors.

**Figure 4:**
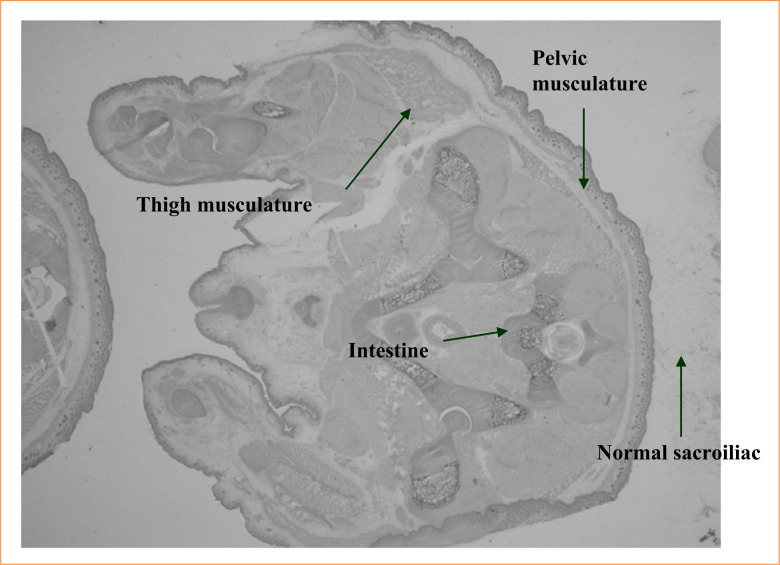
Histological cross-section dyed with hematoxylin and eosin.

Source: Elaborated by the authors.

The pregnant rats were randomly divided into two groups: control (four rats), and experimental (26 rats). Experimental rats received 10% ETU (130 mg/kg) by gavage on gestational day 10. Control rats received the same volume of vehicle only. Rats were kept individually in an air-conditioned, 12-hour light-dark cycle animal laboratory (School of Medicine, Ribeirão Preto, SP, Brazil) and fed with normal rat chow and tap water *ad libitum*. Dams were killed with an overdose inhalation of diethyl ether on gestational day 21. Their embryos were recovered by cesarean section and divided into three groups: the control, the affected (fetuses without the anomaly, but submitted to drug effect), and the malformed (fetuses with the anomaly). Up to four fetuses from each group were randomly selected and frozen in TBS-TFM crioprotector. The fetuses were cross-sectioned in a cryostat with a width of 20 µm. Cross-sections were mounted on previously gelatinized glass slides, leaving a distance of 200 µm for each section. Slides 2 and 3 were used in the study (Fig. 2). Motor neuron plates were identified by immunohistochemistry with acetylcholinesterase (Fig. 3). Musculature and sacroiliac alterations were evaluated by histological and morphometric studies with hematoxylin and eosin (Fig. 4). 

## Results

**Figure 5:**
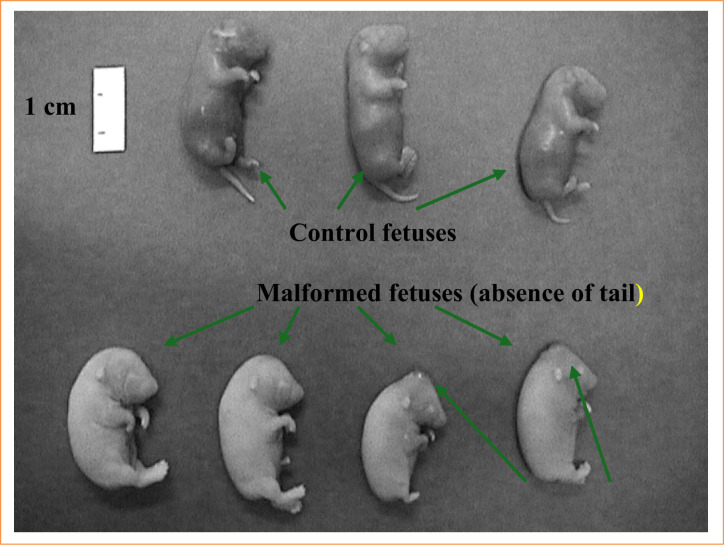
Control and malformed fetuses.

Source: Elaborated by the authors.

Sixty female rats were used in the study; 40 were time-mated (66.6%), and pregnancy was detected in 30 (50%). Four pregnant rats were selected for the control group, and they generated 43 fetuses (average of 10.75 fetuses/female rat). Twenty-six pregnant rats were given ETU, and they generated 200 fetuses (average of 7.7 fetuses/female rat). Among the fetuses exposed to the drug, 82 did not exhibit anorectal anomaly (the affected), whereas 118 had the malformation (the malformed) (Fig. 5). The most frequent associated macroscopic malformations were spina bifida (55 fetuses), encephalocele (20), and alterations in the lower limbs (5). The sacroiliac was malformed in 45% of the affected rats and in 53.2% of the affected rats and in 53.2% of the malformed rats.

The motor neurons were studied by using the ratio number of motor plates / muscular area and enteric neurons considering the number of points visualized per photographic field, for each cross-section (Table 1).

**Table 1:** Results obtained with the immunohistochemistry and histological studies.

	A				B				C				D		
	C	A	MF		C	A	MF		C	A	MF		C	A	MF
Motor plates / muscular area (× 10^-6^)	2,567 / sd 1,163	1,007 / sd 874*	530 / sd 1,044*		1,594 / sd 623	1,319 / sd 984	598 / sd 560*		1,438 / sd 534	845 / sd 228*	563 / sd 384*		1,526 / sd 549	862 / sd 348*	592 / sd 315*
ENS/field	-	-	-		7.1 / sd 7.4	6.9 / sd 8.2	0.06 / sd 0.3*		14.3 / sd 5.9	18.4 / sd 7.5	6.5 / sd 8.9*		19.8 / sd 5.6	22.6 / sd 10.0	14.9 / sd 8.3

* Statistically different from the control group, *p* ≤ 0.05.

Source: Elaborated by the authors.

## Discussion

Analysis of the musculature did not yield statistically different values for the upper cross-sections (B, C, and D). However, in the case of cross-section A, the affected fetuses presented a statistically larger muscular area than the malformed rats.

In 1989, Inomata et al.^11^ reported a direct relationship between sacral alterations and the faulty development of the pelvian musculature. In 1987, Peña^12^ related the sacral anomaly with cases of worse prognosis. In this work, though, values of muscular areas obtained from both the malformed and affected fetuses, with either normal or malformed sacroiliac, are very similar.

Immunohistochemistry studies gave evidence of a lower number of motor plates in the malformed rats, in all cross-sections. However, values obtained for the affected rats are close to those encountered for the control rats.

Analysis of the number of nervous terminations / muscular area showed that there is a statistical difference between the affected and the malformed fetuses in all cross-sections, when compared to the control rats. In the case of cross-section B, though, there is no difference between the control and the affected rats. Peña^12^ has reported a decrease in the number of neurons in the median ventral horn, a value corresponding to the inervation of the muscular complex, in children with severe anorectal anomaly. On the other hand, in 2002 Bitoh et al.^13^ reported a study of rats with retinoic acid-induced anorectal anomaly and stated that alterations in the neural tube and its derived structures take place before the muscular alterations. In 2002, Kubota and Suita^14^ described alterations in the control of the external anal sphincter as being due to a congenital defect, and not to lesions generated during the surgery.

Analysis of the ratio number of motor plates/muscular area in the affected rats with either normal or malformed sacroiliac showed there is no difference between the two groups. The same is observed in the case of the malformed rats with either normal or malformed sacroiliac. Yuan et al.^15^ described a decrease in the number of motor neurons of the muscular complex as a primary alteration that coexists with anorectal anomaly during abnormal fetus development. These authors also reported that fetuses without anorectal anomaly or alterations in the neural tube (affected rat with normal sacroiliac) submitted to drug effect do not show any difference in the distribution and number of motor neurons when compared to the control group. On the other hand, fetuses with anorectal anomaly, with or without alterations in the neural tube (malformed rats with either normal or altered sacroiliac), and fetuses with alterations in the neural tube (affected rats with malformed sacroiliac), exhibit a lower number of neurons which were not only smaller, but also morphologically altered^15^. Moreover, according to Yuan et al.^15^, a reduced number of motor neurons in malformed fetuses with normal sacroiliac suggests that sacral alterations are not the only ones responsible for such reduction. In 1993, Li et al.^16^ also reported reduction in the number of motor neurons in the lateral ventral horn in cases of anorectal anomaly, but they stated that this decrease would only have consequences, such as alterations in sphincter control, if losses amounted to 96 %^16^.

Concerning the enteric inervation, cross-section A was not considered in our analysis because the control group was not stained.

The absence of enteric nervous system staining in cross-section B of the malformed group can be explained as being due to anorectal anomaly (absence of anus), and the hypoplasia observed in the other cross-sections, when compared to the control group. There was no statistically significant reduction in any of the cross-sections of the affected group, when compared to the control rats. In 2002, Meier-Ruge and Holschneider^17^ concluded that there is no correlation between alterations in the enteric nervous system and the type of fistula, but they found out that there is some alteration in the enteric nervous system in 60% of the cases of anorectal anomaly. On the other hand, Martucciello et al.^18^ found intestinal neural dysplasia associated with anorectal anomaly in 20.9% of the cases.

Analysis of the affected rats with either malformed or normal sacroiliac did not give evidence of any statistical difference between them. The same was observed in the case of the malformed rats with either malformed or altered sacroiliac.

The present study has shown there is a reduction in the number of motor neurons of the pelvian musculature of rats with ETU-induced anorectal anomaly and of rats with no evident anorectal anomaly, but submitted to drug effect. The enteric inervation is altered in fetuses with anorectal anomaly, but not in the apparently normal fetuses submitted to the drug. When present, the sacral anomaly did not interfere with the results when a comparison between rats with normal sacroiliac and those with altered sacroiliac was carried out in the same group; that is, in the affected or the malformed groups.

Factors that trigger anorectal anomalies and alterations that lead to poor sphincter control still need to be further investigated, because there is no agreement on the triad muscle-innervation-anorectal anomaly with respect to the sequence of alterations in these structures^19^ ^,20^.

## Conclusion

ETU leads to a reduced number of motor neurons in the pelvic musculature of both the malformed and the affected rats. The enteric neurons are altered in the malformed fetuses, but not in the affected ones. Both the affected and malformed rats exhibit sacral alterations that do not interfere with neurons.

New studies focusing on embryo development from a muscular and nervous viewpoint are necessary to better clarify the alterations encountered in anorectal anomaly.

## Data Availability

All data sets were generated or analyzed in the current study.
